# A Literature Review to Identify Effective Web- and App-Based mHealth Interventions for Stress Management at Work

**DOI:** 10.1177/21650799231170872

**Published:** 2023-05-30

**Authors:** Selina Marita Egger, Sara Frey, Lena Sauerzopf, Ursula Meidert

**Affiliations:** 1 Institute of Occupational Therapy, School of Health Sciences, Zurich University of Applied Sciences

**Keywords:** mHealth, stress, intervention, app, web-based

## Abstract

**Background:**

Persistent job-related stress can be harmful to physical and mental health and has a sizable financial burden on society. Face-to-face interventions are effective in reducing stress but have the disadvantage of high costs and time requirements. mHealth solutions may be an effective alternative to provide stress management interventions at work. Occupational health professionals need information on which mHealth apps are effective for employees to manage job-related stress. The aim of this review is to provide an overview of effective web- and app-based interventions for reduction of job-related stress and stress-related symptoms.

**Method:**

A literature review was conducted in the databases PubMed, PsycINFO, CINAHL Complete, and IEEEXplore.

**Findings:**

A total of 24 articles describing 19 products were found. All products showed effectiveness in trials in improving mental and/or physical health and reducing stress. Most products have a course-like structure with a duration from 1 to 8 weeks. The products use various methods such as psychoeducation and education on stress, cognitive restructuring, emotional regulation, problem-solving, goal setting, gratitude, breathing, or mindfulness techniques. Most products use more than one method and most mixed material such as text on web pages, text messages, videos, reading and audio material, and games.

**Conclusion/Application to Practice:**

Overall, effective mHealth products were identified for the intervention of acute and chronic stress. Occupational health practitioners can use these 19 evidence-based mHealth products when advising organizations on health promotion of employees to reduce stress symptoms and promote health and well-being.

## Background

On a world ranking of employees experiencing work stress on a daily basis in 2021, North America is the second most affected region in the world with 50% of the employees experiencing stress at work. Only East Asia has a higher ranking with 55% of employees facing work stress on a daily basis (Clifton, 2022). Work-related stress increased in many countries through the COVID-19 pandemic (Jewell et al., 2020; Leclerc et al., 2022; McGinty et al., 2020). Some employees were forced to work remotely, and the related social isolation and family–work conflicts were factors that increased stress for numerous employees (Galanti et al., 2021; Xiong et al., 2020). The World Health Organization (2020) defines work stress as the “response people may have when presented with work demands and pressures that are not matched to their knowledge and abilities and which challenge their ability to cope.” Stress becomes dangerous if one does not respond or cope with it adequately (O’Connor et al., 2021), resulting in physical, emotional, and behavioral consequences such as reactions of the nervous, cardiovascular, or respiratory systems, headaches, sleeping disorders, anxiety, fear, sadness, or depression (American Psychological Association [APA], 2021; Yaribeygi et al., 2017). Rehabilitation of diseases caused by stress can be challenging and lengthy for the individual (Korhonen et al., 2020), and stress is a common reason for sick leave (Duchemin et al., 2019). Furthermore, stressed people at work are more likely to leave their job (Omar et al., 2020). Therefore, stress management is a concern for companies, insurers and society, and evidence-based interventions to reduce stress are needed. Stress treatment should be provided promptly to prevent long-lasting or chronic stress and it consequences (APA, 2021; Dai et al., 2020).

mHealth (mobile health) is defined as a mobile wireless technology for the cost-effective and secure use of information and communication technologies in support of health and health-related fields (World Health Organization, 2018). The use of mHealth products has several benefits compared with traditional therapy forms: for example, economically beneficial, the inclusion of undersupplied population groups, possibility to overcome distances, fast access, low usage threshold, anonymity, flexibility in application (Iribarren et al., 2017). Web- and app-based interventions promote and support health and augment conventional health services. In 2021, 350 000 mHealth apps were available in app stores (Byambasuren, 2020). This very large number of apps makes it difficult for users to find and choose an effective and evidence-based product. Byambasuren (2020) states a lack of knowledge of effective mHealth apps and suggests: “a curated compilation of effective [mHealth] apps” (p. 4). To our knowledge, no scientific work provides an overview of mHealth products that have shown to effectively reduce stress and stress related symptoms.

The aim of this literature review, therefore, is to explore which web- and app-based mHealth interventions are effective in stress management at work.

## Methods

**Figure 1.**
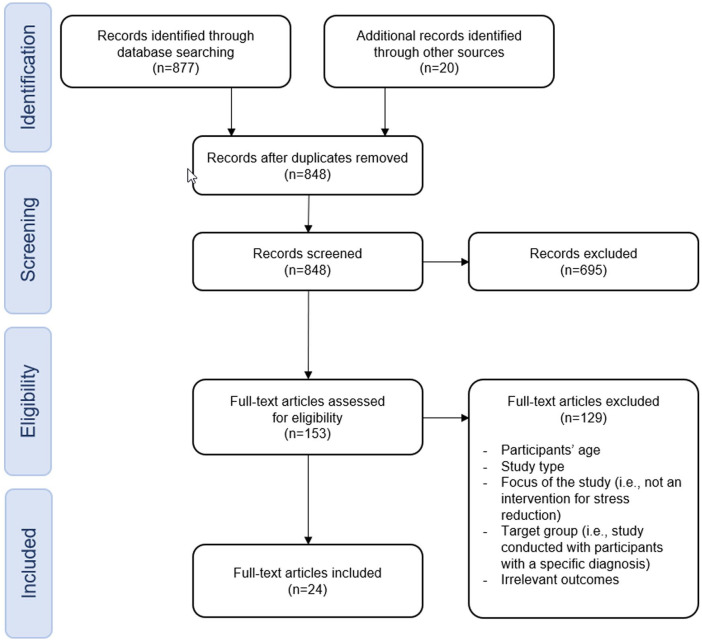
PRISMA Flowchart.

A literature review was performed according to the PRISMA flow diagram (Figure 1). The search was carried out in four scientific databases: PubMed, PsycINFO and CINAHL Complete, and IEEEXplore. The search terms were web-based, stress reduction, intervention, evidence and a broad set of synonyms were used to maximize sensitivity. Boolean operators were used, “OR” within topics and “AND” to combine topics. The search was limited to publication years 2015 to 2022, including working adults (18 years or above) who had no specific diagnosis and to articles written in English or German. Studies were included if they were randomized controlled trials, systematic reviews, or meta-analyses.

The search resulted in a total of 877 articles (see Figure 1). Using Wohlin et al. (2022) snowball method, we identified a further 20 studies by checking the reference list of the articles already found for additional matching articles. After the removal of duplicates, we screened 848 abstracts, whereby 695 articles were excluded because they did not fit the topic or had another focus. In a second step, the full text of the remaining 153 studies was assessed. We excluded 129 records because they did not match our inclusion criteria with regard to study type or target population or because the focus of the study was not an intervention for stress reduction. We ultimately included 24 eligible articles (RCT) in our review.

## Results

We found 24 studies describing 19 products, originating from 7 different countries: Germany (6), Denmark (1), United Kingdom (5), United States (7), Sweden (3), Brazil (1) and Korea (1). Participants in the studies were from a variety of professional backgrounds including pharmaceutical, high-tech, information technology, management (9), hospital (4), university or school (3), or psychologist (1). Seven of the studies included participants from the general working population without being specified.

Of the 19 products, 11 are web-based and eight are apps (see Tables 1 and 2 for summaries of products). All products showed effectiveness. They all improved mental health by decreasing stress-related symptoms such as perceived stress, perceived burnout symptoms, depressive symptoms, emotional exhaustion, and anxiety. Furthermore, improved mental health outcomes were improved resilience (Champion et al., 2018; Kim et al., 2018; Litvin et al., 2020), work commitment (Möltner et al., 2018), job satisfaction (Hersch et al., 2016; Möltner et al., 2018), emotional intelligence (Möltner et al., 2018), and self-efficacy (Cook et al., 2015). Studies of 11 products used stress as primary or secondary outcome measure, quantified by the Perceived Stress Scale of Cohen et al. (1983). Other studies used, for example, stress scales of Orioli (Orioli et al., 1987, as cited in Cook et al., 2015), the Stress Overload Scale (Amirkhan et al., 2015; as cited in Economides et al., 2018), or the Nursing Stress Scale (Gray-Toft & Anderson, 1981 as cited in Hersch et al., 2016). The pre- and post-tests of most studies varied between 2 weeks and 6 months.

**Table 1.** Summary of Studies: No., Study Reference, Study Design, Outcome Categories and Evidence of Effectiveness

No.	Study reference	Study design	Outcome categories	Evidence of effectiveness
1	Möltner, H., Leve, J., & Esch, T. (2018). Burnout-Prävention und mobile Achtsamkeit: Evaluation eines appbasierten Gesundheitstrainings bei Berufstätigen. *Das Gesundheitswesen, 57*(3), 295–300. https://doi.org/10.1055/s-0043-114004	Randomized controlled trial	Mindfulness, work commitment, job satisfaction, innovation and creativity, emotional intelligence, emotional exhaustion.	After 2 weeks, significant differences in pre- and posttest for all scales excluding innovation and creativity in intervention group. Significant differences in post-test between intervention and waiting group.
2	Bennike, I. H., Wieghorst, A., & Kirk, U. (2017). Online-based mindfulness training reduces behavioral markers of mind wandering. *Journal of Cognitive Enhancement, 1*(2), 172–181. https://doi.org/10.1007/s41465-017-0020-9	Randomized controlled trial	Mind wandering, Dispositional mindfulness.	After 4 weeks increase in mindfulness and a reduction in mind wandering.
3	Bostock, S., Crosswell, A. D., Prather, A. A., & Steptoe, A. (2019). Mindfulness on-the-go: Effects of a mindfulness meditation app on work stress and well-being. *Journal of Occupational Health Psychology, 24*(1), 127–138. https://doi.org/10.1037/ocp0000118	Randomized controlled trial	Well-being, Psychological distress, Job strain, Workplace social support, Mindfulness, blood pressure.	After 8 weeks improved well-being, decreased distress in working, improved workplace social support, job strain, depressive symptoms, and anxiety.
4	Champion, L., Economides, M., & Chandler, C. (2018). The efficacy of a brief app-based mindfulness intervention on psychosocial outcomes in healthy adults: A pilot randomized controlled trial. *PLOS ONE, 13*(12), Article e0209482. https://doi.org/10.1371/journal.pone.0209482	Randomized controlled trial Pilot	Satisfaction with Life Scale, Perceived Stress Scale, Wagnild Resilience Scale.	Increased psychosocial well-being over a period of 10 or 30 days.
5	Economides, M., Martman, J., Bell, M. J., & Sanderson, B. (2018). Improvements in stress, affect, and irritability following brief use of a mindfulness-based smartphone app: A randomized controlled trial. *Mindfulness, 9*(5), 1584–1593. https://doi.org/10.1007/s12671-018-0905-4	Randomized controlled trial	Stress Overload, Positive and negative affect, frustration, and irritability.	Significant impact on irritability, affect, and stress.
6	Howells, A., Ivtzan, I., & Eiroa-Orosa, F. J. (2016). Putting the “app” in Happiness: A randomized controlled trial of a smartphone-based mindfulness intervention to enhance wellbeing. *Journal of Happiness Studies, 17*(1), 163–185. https://doi.org/10.1007/s10902-014-9589-1	Randomized controlled trial	Satisfaction with Life, well-being, positive and negative feelings, de-pressive symptoms.	After 10 days, significant improvements in positive affect and depression (Howells et al., 2016).
7	Querstret, D., Cropley, M., & Fife-Schaw, C. (2017). Internet-based instructor-led mindfulness for work-related rumination, fatigue, and sleep: Assessing facets of mindfulness as mechanisms of change. A randomized waitlist control trial. *Journal of Occupational Health Psychology, 22*(2), 153–169. https://doi.org/10.1037/ocp0000028	Randomized controlled trial	Work-Related Rumination, Work-related fatigue, Sleep quality, Mindfulness.	Significantly lowering levels of affective rumination, problem-solving pondering, chronic and acute fatigue, significantly improved sleep quality, when compared with participants not completing the course. Maintaining effects after 3- and 6-month follow-up.
8	Heber, E., Lehr, D., Ebert, D. D., Berking, M., & Riper, H. (2016). Web-based and mobile stress management intervention for employees: A randomized controlled trial. *Journal of Medical Internet Research, 18*(1), e21. https://doi.org/10.2196/jmir.5112	Randomized controlled trial	Primary outcome: Perceived stress Secondary outcome: Mental health, work-related health, Emotion regulation, client satisfaction.	After 7 weeks and 6 months, effectively reduces symptoms of perceived stress. Beneficial effects on depression, anxiety, worrying, insomnia severity, mental health component of quality of life, work-related health (i.e., emotional exhaustion, psychological detachment from work) and skill-related (general emotion regulation skills, emotion specific emotional regulation skill for general distress) outcomes. No effects on absenteeism, presenteeism, work engagement and the physical component of quality of life.
9	Ebert, D. D., Heber, E., Berking, M., Riper, H., Cuijpers, P., Funk, B., & Lehr, D. (2016). Self-guided internet-based and mobile-based stress management for employees: Results of a randomized controlled trial. *Occupational and Environmental Medicine, 73*(5), 315–323. https://doi.org/10.1136/oemed-2015-103269	Randomized controlled trial	Primary outcome: Perceived stress Secondary outcome: Mental health, work-related health, skills/ competencies, demographic variables and client satisfaction.	7 weeks and 6 months lower scores on perceived stress scale post-test and 6-month follow-up as compared to the control group. A large effect size was observed at post-test. The effect was moderate to large at the 6-month follow-up. Significant effects in mental health-related outcomes, work-related health, skills, and competencies
10	Hersch, R. K., Cook, R. F., Deitz, D. K., Kaplan, S., Hughes, D., Friesen, M. A., & Vezina, M. (2016). Reducing nurses’ stress: A randomized controlled trial of a web-based stress management program for nurses. *Applied Nursing Research, 32*, 18–25. https://doi.org/10.1016/j.apnr.2016.04.003	Randomized controlled trial	Nursing stress scale, symptoms of distress, coping with stress, work limitations, use of substances for stress relief, dinging quantity and frequency, depression and anxiety, job satisfaction.	After 3 months, significantly greater improvement in the measure of nurses’ stress in all subscales of the nursing stress scale except the subscale lack of support. No significant outcomes for distress, coping with stress, work limitation, nurses’ job satisfaction, understanding depression and anxiety, using substances to relieve stress, or alcohol quantity and frequency.
11	Eriksson, T., Germundsjö, L., Åström, E., & Rönnlund, M. (2018). Mindful self-compassion training reduces stress and burnout symptoms among practicing psychologists: A randomized controlled trial of a brief web-based intervention. *Frontiers in Psychology, 9*. https://doi.org/10.3389/fpsyg.2018.02340	Randomized controlled trial	Self-compassion, mindfulness, perceived stress, burnout.	After 6 weeks Increased self-compassion, improved mindfulness skills, significant reductions in perceived stress and burnout symptoms.
12	Stjernswärd, S., & Hansson, L. (2017). Effectiveness and usability of a web-based mindfulness intervention for families living with mental illness. *Mindfulness, 8*(3), 751–764. https://doi.org/10.1007/s12671-016-0653-2	Randomized controlled trial	Mindfulness, caregiver burden, self-compassion, perceived stress, usability, confounding factors and negative effects of training.	After 8 weeks, significant improvements in mindfulness, self-compassion, perceived stress and three of seven dimensions of the caregiver burden (relational problems, mental health, and problems with daily activities). After 3 months improvements in fulfillment and relational problems.
13	Allexandre, D., Bernstein, A. M., Walker, E., Hunter, J., Roizen, M. F., & Morledge, T. J. (2016). A web-based mindfulness stress management program in a corporate call center. *Journal of Occupational and Environmental Medicine, 58*(3), 254–264. https://doi.org/10.1097/JOM.0000000000000680	Randomized controlled trial	Perceived stress, burnout, mindfulness, psychological and emotional well-being, and work performance.	Significant improvements from baseline to 8 weeks in all outcome measures except productivity and professional efficacy. Improvements were maintained or increased at 16 weeks in all the intervention groups, except for emotional exhaustion, professional efficacy and mindfulness. The intent-to-treat analysis, showed that participants with and without group support had greater reductions from baseline to 8 weeks than control for perceived stress, increases in emotional well-being and vitality. These effects persisted at 16 weeks. Participants with group support also showed greater effect than control at 8 weeks for professional efficacy, emotional role functioning, and mindfulness. But not maintained at 16 weeks. Overall, effect sizes in comparison to control were larger for those with group support than for those without.
14	Asplund, R. P., Dagöö, J., Fjellström, I., Niemi, L., Hansson, K., Zeraati, F., Ziuzina, M., Geraedts, A., Ljótsson, B., Carlbring, P., & Andersson, G. (2018). Internet-based stress management for distressed managers: Results from a randomised controlled trial. *Occupational and Environmental Medicine, 75*(2), 105–113. https://doi.org/10.1136/oemed-2017-104458	Randomized controlled trial	Primary outcome: Perceived stress Secondary outcome: Burnout, depression, Insomnia, alcohol use, work-related health, absenteeism, presenteeism and healthcare consumption, utility, and user friendliness.	Significantly lower levels of perceived stress, burnout, depression, and insomnia severity, at post-treatment. Significantly higher satisfaction with work, with regard to support, internal work experience, management, and process of change. 6 months follow-up, both groups were relatively stable or continued to improve on the primary and secondary outcome measures.
15	Cook, R. F., Hersch, R. K., Schlossberg, D., & Leaf, S. L. (2015). A web-based health promotion program for older workers: Randomized controlled trial. *Journal of Medical Internet Research, 17*(3), e82. https://doi.org/10.2196/jmir.3399	Randomized controlled trial	Distress, coping with stress, diet outcome expectancies, barriers to a healthy diet, eating practices, over-eating self-efficacy, diet change self-efficacy, planning healthy eating, weight and body mass index, exercise habits, exercise self-efficacy, self-efficacy for overcoming barriers to exercise, exercise planning, beliefs about aging, tobacco use.	After 3 months, a significant greater improvement in diet behavioral change self-efficacy, planning healthy eating, and mild exercise. No significant program effects on measures of stress or aging beliefs.
16	Domes, G., Stächele, T., von Dawans, B., & Heinrichs, M. (2019). Effects of internet-based stress management on acute cortisol stress reactivity: Preliminary evidence using the Trier Social Stress Test for Groups (TSST-G). *Psychoneuroendocrinology, 105*, 117–122. https://doi.org/10.1016/j.psyneuen.2018.12.001	Randomized controlled trial Pilot	Stress, saliva sampling and cortisol analysis, and heart rate	After 6 weeks, signiﬁcant reductions in perceived everyday stress. Reduced cortisol response in the stress management group and a trend toward lower cardiovascular reactivity to an acute psycho-social stressor. The groups did not differ in their subjective stress response.
17	Galante, J., Bekkers, M.-J., Mitchell, C., & Gallacher, J. (2016). Loving-kindness meditation effects on well-being and altruism: A mixed-methods online RCT. *Applied Psychology: Health and Well-Being, 8*(3), 322–350. https://doi.org/10.1111/aphw.12074	mixed-method Randomized controlled trial	Primary outcome: well-being Secondary outcome: satisfaction with life, pleasant emotions, coping with life events, outward and inward irritability, perceived stress, anxiety, absence of physical discomfort.	After 4 weeks, reduce anxiety and stimulate altruism. Greater increase in perspective-taking and more helping behavior but not enough to reach signiﬁcance. There is no evidence that Loving-kindness training (LKM) is more effective than light physical exercise training (LE) in improving well-being and reducing stress. Both led to stress reduction and greater well-being.
18	Jonas, B., Leuschner, F., & Tossmann, P. (2017). Efficacy of an internet-based intervention for burnout: A randomized controlled trial in the German working population. *Anxiety, Stress, & Coping, 30*(2), 133–144. https://doi.org/10.1080/10615806.2016.1233324	Randomized controlled trial	Emotional exhaustion, cynicism, professional efficacy, depression, anxiety, stress, physical well-being, psychological well-being.	After 3 months, the intervention group had significantly lower depression rates, were less cynical about their profession, and had a higher sense of personal accomplishment. Improvements in exhaustion, stress, anxiety, and psychological well-being but not statistically significant.
19	Coelhoso, C. C., Tobo, P. R., Lacerda, S. S., Lima, A. H., Barrichello, C. R. C., Amaro, E., Jr., & Kozasa, E. H. (2019). A new mental health mobile app for well-being and stress reduction in working women: Randomized controlled trial. *Journal of Medical Internet Research, 21*(11), e14269. https://doi.org/10.2196/14269	Randomized controlled trial	Primary outcome: Perceived stress, well-being, subjective symptoms of stress and well-being during the last month. Secondary outcomes: subjective symptoms of stress and well-being at the moment.	After 4 and 8 weeks, significant decreases in perceived stress, increase in well-being, improvements in work-related stress, greater reduction in general stress, work-related well-being increased significantly.
20	Smith, E. N., Santoro, E., Moraveji, N., Susi, M., & Crum, A. J. (2020). Integrating wearables in stress management interventions: Promising evidence from a randomized trial. *International Journal of Stress Management, 27*(2), 172–182. https://doi.org/10.1037/str0000137	Randomized controlled trial	Primary outcome: perceived stress, mood and anxiety symptoms, physical and mental health over time. Secondary outcome: well-being, quality of life, positive and negative affect, engagement and fidelity, respiratory data.	After 4 weeks, treatment group reported 15.8% fewer negative instances of stress, 13.0% fewer distressing symptoms, and 28.2% fewer days feeling anxious or stressed compared with a waitlist control. Furthermore, the treatment and waitlist control groups reported similar amounts of stress experienced. This suggests that participants in the treatment group did not simply avoid challenges or stress; they found ways to manage the same amount of stress in more effective ways.
21	Kim, J. I., Yun, J.-Y., Park, H., Park, S.-Y., Ahn, Y., Lee, H., Kim, T.-K., Yoon, S., Lee, Y.-J., Oh, S., Denninger, J. W., Kim, B.-N., & Kim, J.-H. (2018). A mobile videoconference-based intervention on stress reduction and resilience enhancement in employees: Randomized controlled trial. *Journal of Medical Internet Research, 20*(10), e10760. https://doi.org/10.2196/10760	Randomized controlled trial	Perceived stress, emotional labor, occupational stress, resilience, insomnia.	After 4 weeks and 1 month, significantly differential effects across time according to treatment condition on perceived stress, resilience, emotional labor, and sleep. No significant differences between the mobile videoconferencing and in-person conditions at follow-up.
22	Hirshberg, M. J., Frye, C., Dahl, C. J., Riordan, K. M., Vack, N. J., Sachs, J., Goldman, R., Davidson, R. J., & Goldberg, S. B. (2022). A randomized controlled trial of a smartphone-based well-being training in public school system employees during the COVID-19 pandemic. *Journal of Educational Psychology*. https://doi.org/10.1037/edu0000739	Randomized controlled trial	Primary outcome: psychological distress Secondary outcomes: global well-being, perseverative thinking, awareness (mindful action), connection (lone-liness/ social connection and self-compassion), insight (cognitive defusion or the ability to be aware of but not fused with experience), purpose (meaning in life).	After 4 weeks effective in reducing psychological distress at post-intervention and as well at 3-month follow-up. Similar indications of immediate and sustained benefit of the experimental group were found for secondary outcomes.
23	Chelidoni, O., Plans, D., Ponzo, S., Morelli, D., & Cropley, M. (2020). Exploring the effects of a brief biofeedback breathing session delivered through the BioBase app in facilitating employee stress recovery: Randomized experimental study. *JMIR MHealth and UHealth, 8*(10), e19412. https://doi.org/10.2196/19412	Randomized experimental trial	Heart rate variability, mindfulness, fatigue, sleepiness, mood.	Performing BioBase breathing showed significant higher heart rate variability during recovery period after stress induction compared with the mindfulness group and control group.
24	Litvin, S., Saunders, R., Maier, M. A., & Lüttke, S. (2020). Gamification as an approach to improve resilience and reduce attrition in mobile mental health interventions: A randomized controlled trial. *PLOS ONE, 15*(9), Article e0237220. https://doi.org/10.1371/journal.pone.0237220	Randomized controlled trial	Well-being (measured by different constructs related to well-being: resilience, personal growth, interpersonal relationship skills, anxiety). Primary outcome: resilience. Secondary outcomes: personal growth, interpersonal relationship skills, anxiety.	After 5 weeks, significantly increased resilience was found in the intervention group compared with the control and waiting list group. Furthermore, also secondary outcomes showed significant positive changes, presenting increase personal growth and positive relations with other as well as decreased self-reported anxiety.

**Table 2.** Summary of Products: Product name, Nr., Product Type, Product Validation, Technique and Conceptual Framework, and Short Description of Product

Product name	No. (Table 1)	Product type	Product validation	Technique and conceptual framework	Short description of product
Be mindful	7	Web-based *(Available in English)*	n.a.	Meditation Elements of mindfulness-based stress reduction (MBSR; Kabat-Zinn, 1990) and mindful-ness-based cognitive therapy (MBCT; Teasdale et al., 2000).	Website with instructional videos and audio files to guide formal meditations for work-related rumination (affective rumination, problem-solving pondering), fatigue (acute fatigue, chronic fatigue) and improved sleep quality.
Beratung hilft’ (counseling helps)	18	Web-based *(Available in German)*	n.a.	Life-chat, stress management strategies. Personal communication within the intervention follows a solution-focused approach and centers on the clients’ strengths, resources and goals (De Shazer, 2005). Exercise modules are based on CBT, trying to positively influence affect and behavior through cognitive re-structuring (Beck, 2011)	Structured and therapist-guided, online-intervention for burnout and work-related stress. The intervention comprises both synchronous communication via live-chat and asynchronous communication (weekly feedback).
BioBase	23	App *(Available in English on the App Store)*	n.a.	Breathing training	BioBase is a brief app-based breathing intervention with the aim to facilitate stress recovery among employees. The app provides guided breathing, and it can be modified according to the users’ heart rate.
eQuoo	24	App *Available in English on the App Store and Google Play Store*	n.a.	Cognitive behavioral therapy, positive psychology therapies, systemic therapies	The app contains five levels that are intended to be used over a period of 5 weeks. Within these levels, different psychological skills are taught in form of tutorials. These tutorials lead the user across a series of real-life scenarios. Furthermore, this gamified app confronts the user with some challenges presented in different genres.
Florescer	19	App *Available in Portuguese*	n.a.	Psychoeducation and emotion management strategies Mindfulness based on positive psychology	Mobile app training designed to handle psychological stress based on relaxation training, breathing techniques, meditation (such as mindfulness, loving, kindness, and empathetic joy), and positive psychology principles.
GET.ON Stress	8,9	Web-based *Available in German*	96.4% rated the program quality excellent/ good.	Problem solving, emotion regulation. Lazarus’ transactional model of stress Problem-focused coping through the use of cognitive or behavioral efforts.	Website with general information, examples related to work, interactive exercises, quizzes, audio and video files, work sheets and mp3 files, short daily stress diary, homework, e-coach applying an adherence-focused guidance concept, consisted of two elements: (a) adherence monitoring and (b) feedback on demand.
Headspace: Meditation & Sleep	2-6	App *(App Store and Google Play Store)*	Rated as number one mindfulness app of 23 apps based on criteria including engagement, functionality, visual aesthetics, and information quality.	Meditation Mindfulness Psychoeducation	Meditation app with guided meditation on various topics. *Available in German, English, French, Portuguese, Spanish on the*
HMP	22	App *(Available in English, Spanish on the App Store and Google Play Store)*	n.a.	Meditation, Psychoeducation	The HMP is a self-guided, meditation-based well-being training. The app provides different kinds of meditations as well as brief pod-casts with key insights from scientific research on well-being.
Hello Mindcare	21	App (Available in Korean)	n.a.	Life-Therapy, Stress Management Techniques Cognitive behavioral therapy and positive psychology in conjunction with methods that elicit a relaxation response.	Hello Mindcare Android App allows direct peer-to-peer communication. App provides booking system, videoconferencing, document sharing, workbooks.
IBSM	16	Web-based *(Available in German)*	n.a.	Psycho-Education and stress management strategies IBSM represents an interactive, electronic adaptation of face-to-face cognitive-behavioral stress-management training sessions, such as stress inoculation training by Meichenbaum (1985).	short-term intervention to improve relevant skills for the management of daily stressful situations, particularly in occupational settings. Video clips, audio files, animations, and interactive elements.
iSMI	14	Web-based *(Available in Swedish)*	On a 7-point scale (1=low satisfaction; 7=high satisfaction). Utility was given an average of 5.8 for all eight modules/ weeks (*SD* = 0.67; range = 5.5–5.8) and user-friendliness, 5.1 (*SD* = 0.96; range = 4.8–6.0). Modules on exposure in different stress-related areas (e.g., assertive-ness, perfectionism and procrastination) and feedback received the highest rating and physical exercise and applied relaxation was rated lowest on both utility and user-friendliness.	Psychoeducation and stress management strategies. Acceptance Commitment Therapy and behavioral activation, CBT, third wave CBT.	Each module contained text, exercises, worksheets, images, examples, audio-files and video-files and homework exercises.
Loving kindness meditation	17	Web-based *(Available in English)*	A pilot study was conducted prior to the trial to test the website’s functionality and the feasibility and acceptability of the Internet-based LKM course.	Loving kindness meditation based on popular LKM literature (mainly, and with permission, Salzberg, 1995)	Online video and reminder email, electronic diary, and forum.
Mindfulness and compassion with self and others	11	Web-based *(Available in Swedish)*	n.a.	Mindfulness intervention	Initial instruction video: 6 steps involving different types of exercises-guided instructions (auditory files): kind attention, kind awareness, loving kindness with oneself and others, self-compassion part 1 and 2, compassion with others and quiet practice, online diary.
Mindfulness program	12	Web-based *(Available in Swedish)*	Intervention showed good acceptance, feasibility, usability, and user value, with ease of use and convenience of access representing strong motivators for use (Stjernswärd & Hansson, 2016)	Mindfulness intervention	Program designed for families of mentally ill people, related to carer’s situation and associated experiences of burden and stress. Audio/video files (960 min) accompanied by written keywords on the screen, descriptive text files and instructions for daily mind-fulness exercises inclusive (self) compassion exercises, time log a private diary. Weekly e-mail reminders, including contact information to the research group/ technical support.
Spire Stone	20	App and wearable *(Available in English on Google Play Store)*	86% of participants re-ported they would be at least somewhat likely to recommend the stone device to a friend and 17% would be very likely (incl. a 9 or 10 on the scale) to recommend the device.	Breathing training, Mindfulness	In-app mindfulness-based breathing sessions (Boosts), Wearing the Spire Stone (Wearable), In-app historical log of physiological states. In-app bio-feedback of cur-rent physiology. Realtime notifications of physiological states.
Stress Free Now	13	Web-based *(Available in English, German, Hungarian, Romanian)*	n.a.	Mindfulness	Interactive, educational program for stress reduction based on mindfulness meditation.
the BREATHE	10	Web-based *(Available in English)*	The program was developed through a series of steps which included focus groups with nurses for the look, feel, and content of the program; discussions with experts in the ﬁeld; and content review and reﬁnement among the project staff and the technical development staff.	Psychoeducation and stress management strategies 2 major theories of health behavior change social-cognitive theory (SCT) and stages-of-change theory. Conceptual model articulated by Cook and Youngblood.	Program designed for nurses. Includes sections on how stress impacts the body; assessing stress and identifying stressors; practical stress management tools addressing changing one’s views of stressors, changing one’s response to stressors, or changing the stressful situation; promoting effective communication skills; taking time to grieve; and depression and anxiety. It includes interactive exercises, downloadable tools, real story videos from nurses, and other audio/visual content.
Web-based HealthyPast 50 program	15	Web-based *(Available in English)*	With the exception of the module on healthy aging, all modules incorporated material from previously developed and tested programs from our group (for which evidence of efficacy was shown), modifying the content and approaches for the 50 and older audience.	Psychoeducation and stress management strategies. Social-cognitive conceptual model based primarily on the work of Bandura [24-25], emphasizing the boosting of self-efficacy, self-regulation, and planning.	Web-based multimedia program containing information and guidance on the major health pro-motion topics. Text, audio, video, graphics, and narration.
7Mind Meditation and Mindful-ness	1	App *(Available in German, English, French, Dutch on the App Store and Google Play Store)*	n.a.	Meditation Mindfulness	App for guided meditations; thematic courses with meditations against stress, for good sleep, happiness, more concentration, self-confidence, serenity, etc.

*Note.* CBT = Cognitive behavioral therapy; HMP = Healthy Minds Program; IBSM = Internet based stress management program; LKM = Loving-kindness training; iSMI = Guided internet-based stress management intervention.

### Content of Products

Most of the programs include introductions, theoretical, or educational inputs (e.g., concept of meditation and mindfulness, how stress affects the body and daily life, problem-solving methods; for details see Table 2). There are products that cover a variety of health-related topics such as the web-based “HealthyPast 50” program (Cook et al., 2015) or the “GET.ON Stress” program (Ebert et al., 2016). In “HealthyPast 50,” based on an initial assessment, participants receive information on a selection of several health topics such as healthy eating, active lifestyle, tobacco use, weight management, and stress management.

Meditation interventions such as the “Healthy Minds Program” (Hirshberg et al., 2022), the “Be mindful” (Querstret et al., 2017), the “Mindfulness program” (Stjernswärd & Hansson, 2017), or the “7Mind Meditation and mindfulness” (Möltner et al., 2018) contain various methods of meditation rather than focusing solely on stress reduction. Their aim is to improve awareness, well-being, sleep quality, happiness, concentration, and self-confidence. The app “Headspace” covers meditation exercises on topics such as overcoming fear, stress reduction breathing, joy, calmness, and concentration (Bostock et al., 2019; Economides et al., 2018). The focus of the web-based program “mindfulness and compassion with self and others” offers mindfulness exercises as well as compassion-focused exercises regarding loving-kindness and exercises to practice compassion with self and others (Eriksson et al., 2018). The apps “Stress free now” and “BioBase” only comprise breathing training modules based on mindfulness stress reduction (Chelidoni et al., 2020; Smith et al., 2020). “Stress free now” can additionally be combined with a wearable device that provides real-time notifications on significant and sustained changes of respiratory patterns (Smith et al., 2020).

The apps “Florescer” and “Hello Mincare” focus on psychoeducation and education on stress, cognitive restructuring, emotional regulation, problem-solving, goal setting, and gratitude. The web-based program “Get.ON stress” is an intervention based on psychoeducation, including modules regarding problem-solving, emotion regulation, and planning for the future. The optional modules provide information on time management, rumination and worrying, psychological detachment from work, sleep restriction, stimulus control, nutrition, exercise, planning of breaks during work, and social support. The self-guided program includes different interactive elements (e.g., online diary; Ebert et al., 2016). The web-based programs “iSMI” and “the BREATHE” show a similar structure. Both start with an introduction and information about stress followed by coping with work environmental stressors, symptoms of stress, and how to manage those symptoms. The iSMI covers relaxation and cognitive–behavioral therapy principles, exercises to practice work–home interface and value-based action skills (Acceptance Commitment Therapy) as well as practice balance and exposure or modules on positive management using various materials (Asplund et al., 2018). “The BREATHE” provides different stress management strategies and tools and addresses additional topics such as avoiding negative coping (use of alcohol and drugs) and improve mental health (focus on depression and anxiety). These topics are provided through interactive exercises, downloadable tools, and real story videos from nurses or other audio/visual contents. The program includes a tool to track stress (Hersch et al., 2016).

### Different Materials Used to Convey Content

All 19 products use mixed materials. Texts provide instructions or background information on a regular basis. For example, the “stress free now” program (Allexandre et al., 2016) provides daily articles about evidence of each weeks theme or discusses cognitive and behavioral strategies, activities, and concepts supporting or related to mindfulness. Videos allow practicing exercises when needed (Galante et al., 2016). Video introductions give basic information about stress and coping methods (Ebert et al., 2016). Audio material provides guided meditations, body scans, mindful movements instructions, or background information to a specific topic (Asplund et al., 2018; Cook et al., 2015; Domes et al., 2019; Ebert et al., 2016; Heber et al., 2016; Querstret et al., 2017; Stjernswärd & Hansson, 2017). Diaries (e.g., in form of a blog) are used to write down expectations or course experiences or for self-reflection about stress behavior (Galante et al., 2016). To build empathy with the product, the “web-based HealthyPast 50 program” shows photos of elderly people, photos of people at same age as target users. Some programs provide text messages, for example, to remind users to breathe consciously and relax, or to remind them to start the mediation session. Worksheets contain strategies or concepts relating to the interventions. One product also makes use of gamified stress reduction interventions (Litvin et al., 2020).

### Interventions

Most products contain interventions with a modular structure. For example, the intervention in the product “Be mindful” requires the completion of a session before starting a new one (Querstret et al., 2017). The amount of time required to complete the interventions varies between products. There are products such as the “Be mindful” or “Florescer” that are to be performed during a fixed period of 4 to 8 weeks (Coelhoso et al., 2019; Querstret et al., 2017). Others have an unlimited and continuous number of sessions as, for example, “Headspace” with one session per day (Bostock et al., 2019). The duration of the single sessions varies from 3 to 60 min between the products.

## Discussion

To reduce work-related stress and improve stress management, mHealth solutions are an effective and useful alternative to common face-to-face interventions.

Our review provides an overview of mHealth products that reduce perceived stress and stress-related symptoms and increase well-being. There is a great variety in form and content between the 8 apps and 11 web-based interventions we found. Besides stress reduction, various health outcomes are targeted by the interventions and provide manifold options for preventing and addressing stress at work. Especially meditation, mindfulness, or breathing exercises are popular content. Martín et al. (2018), who analyzed mobile apps for stress management, show similar results: Most apps included in their review use relaxing sounds or music and breathing and relaxation exercises.

Some of the mHealth products described in our study are intended to be used in acute stress situations, such as apps with guided meditations or apps combined with a wearable that alerts users when stress occurs and then suggests an appropriate intervention. Other products address more general stress reduction and prevention, for example, to better cope with upcoming stressful situations.

Findings from our review show that mHealth products can be used to effectively address work related stress. However, there are limitations. The rapid development in the market of mHealth products requires a continuous update of an overview of evidence-based products available. A further limitation is that most studies measure outcomes over a period 2 weeks to 6 months. The long-term effect of the products remain unclear. Furthermore, not all products showed effectiveness in every outcome measure. For example, the study by Cook et al. (2015) showed significant improvement in components such as healthy eating and exercises but not in measures regarding stress. Since there is often a correlation between the different outcome measures (e.g., the interaction of nutritional behavior and stress), it can be assumed that this intervention may have a positive impact on stress perception in the long run (El Ansari et al., 2014).

As most products use several methods to address stress reduction, specific success factors of single mHealth intervention cannot be fully determined. Further research is needed to identify key elements for effective mHealth interventions. Furthermore, if study protocols used the same measurement tools such as the Perceived Stress Scale (Cohen et al., 1983), it would facilitate the comparison of the various interventions and products.

Nevertheless, the present literature review provides an overview of effective mHealth interventions for health professionals and for employees. Thereby, mHealth products are an attractive alternative to conventional face-to-face interventions to address stress-related health issues. Especially after the Covid-19 pandemic, working remotely remains popular and remote access to stress interventions is needed. With the expected long-term increase in people working from home, this growth in stress will also be increasingly encountered by Occupational health practitioners (OHP) in their day-to-day work. Online tools will allow for real-time interventions independent of geographic workplace.

## Implications for Occupational Health Nursing Practice or Implications for Occupational Health Practice

Due to the confusing amount of available mHealth products, it can be difficult for OHP’s to find guidance for recommending and prescribing evidence-based products. This review shows effective mHealth products to prevent and treat stress-related symptoms that have the potential for the use in occupational health practice. To promote the use of the products, it can be recommended that different products (e.g. with different materials, interventions and thematic contents) are offered. Thus, employees can choose products that correspond to their preferences.

**Applying Research to Occupational Health Practice**

mHealth products are an attractive alternative to face-to-face interventions for stress reduction and stress-related health issues at work. We identified 19 products that are effective, inexpensive, and flexible with regard to time and location of use. OHP’s can use these 19 evidence-based mHealth products when advising organizations on the health promotion of employees to reduce stress symptoms and promote health and well-being.
